# Road to maternal death: the pooled estimate of maternal near-miss, its primary causes and determinants in Africa: a systematic review and meta-analysis

**DOI:** 10.1186/s12884-024-06325-1

**Published:** 2024-02-17

**Authors:** Aklilu Habte, Habtamu Mellie Bizuayehu, Lire Lemma, Yordanos Sisay

**Affiliations:** 1 School of Public Health, College of Medicine and Health Sciences, Wachemo University, Hosanna, Ethiopia; 2 School of Public Health, Faculty of Medicine, the University of Queensland, Brisbane, Australia; 3 Department of Health Informatics, School of Public Health, College of Medicine and Health Sciences, Wachemo University, Hosanna, Ethiopia; 4 Department of Epidemiology, College of Health Science and Medicine, Wolaita Sodo University, Wolaita Sodo, Ethiopia

**Keywords:** Maternal near-miss, Determinants, Africa, Systematic Review, Meta-Analysis

## Abstract

**Background:**

Maternal near-miss (MNM) is defined by the World Health Organization (WHO) working group as a woman who nearly died but survived a life-threatening condition during pregnancy, childbirth, or within 42 days of termination of pregnancy due to getting quality of care or by chance. Despite the importance of the near-miss concept in enhancing quality of care and maternal health, evidence regarding the prevalence of MNM, its primary causes and its determinants in Africa is sparse; hence, this study aimed to address these gaps.

**Methods:**

A systematic review and meta-analysis of studies published up to October 31, 2023, was conducted. Electronic databases (PubMed/Medline, Scopus, Web of Science, and Directory of Open Access Journals), Google, and Google Scholar were used to search for relevant studies. Studies from any African country that reported the magnitude and/or determinants of MNM using WHO criteria were included. The data were extracted using a Microsoft Excel 2013 spreadsheet and analysed by STATA version 16. Pooled estimates were performed using a random-effects model with the DerSimonian Laired method. The I^2^ test was used to analyze the heterogeneity of the included studies.

**Results:**

Sixty-five studies with 968,555 participants were included. The weighted pooled prevalence of MNM in Africa was 73.64/1000 live births (95% CI: 69.17, 78.11). A high prevalence was found in the Eastern and Western African regions: 114.81/1000 live births (95% CI: 104.94, 123.59) and 78.34/1000 live births (95% CI: 67.23, 89.46), respectively. Severe postpartum hemorrhage and severe hypertension were the leading causes of MNM, accounting for 36.15% (95% CI: 31.32, 40.99) and 27.2% (95% CI: 23.95, 31.09), respectively. Being a rural resident, having a low monthly income, long distance to a health facility, not attending formal education, not receiving ANC, experiencing delays in health service, having a previous history of caesarean section, and having pre-existing medical conditions were found to increase the risk of MNM.

**Conclusion:**

The pooled prevalence of MNM was high in Africa, especially in the eastern and western regions. There were significant variations in the prevalence of MNM across regions and study periods. Strengthening universal access to education and maternal health services, working together to tackle all three delays through community education and awareness campaigns, improving access to transportation and road infrastructure, and improving the quality of care provided at service delivery points are key to reducing MNM, ultimately improving and ensuring maternal health equity.

**Supplementary Information:**

The online version contains supplementary material available at 10.1186/s12884-024-06325-1.

## Background

Despite improvements and worldwide attention on maternal mortality, it is still one of the top global health agendas, and there are many existing challenges to ending preventable maternal mortality, particularly in low and middle-income countries [1]. Successes in lowering maternal mortality during the Millennium Development Goal era have plateaued in the first five years (2016–2020) of the Sustainable Development Goals (SDG) [2]. If this progress is maintained, the Maternal Mortality Ratio (MMR) will be 222 by 2030, more than three times the SDG global target of 70 [2]. Globally, 287,000 maternal deaths occur each year, with Sub-Saharan Africa accounting for 70% of deaths [1].

Many women survive for every woman who dies, yet often experience long-lasting complications, such as adverse pregnancy outcomes, disability, and psychological complications [3, 4]. In 2004, the World Health Organization (WHO) highlighted the importance of moving beyond simply reporting deaths to create an understanding of why they occur and how they might be prevented [5]. Furthermore, in 2011, the concept of maternal near-miss emerged as a tool for assessing the quality of obstetric care [6]. Maternal near-miss (MNM) is defined by the WHO working group as a woman who nearly died but survived a life-threatening condition that occurred during pregnancy, childbirth, or within 42 days following childbirth due to getting the best evidence-based quality care or by chance [5, 7]. Its primary causes are hemorrhage, hypertensive disorders of pregnancy, postpartum sepsis, obstructed labor, uterine rupture, abortion, and anemia [1, 8, 9].

The near-miss approach is comprehensive and works on the concept of criterion-based clinical audit, which is considered a feasible and beneficial method of auditing the quality of maternal health care [10]. It assumes that women who survived life-threatening complications related to pregnancy and childbirth had many similarities with those who died [6]. The ultimate goal of the near-miss approach is to boost clinical practice and reduce preventable morbidity and mortality using the best evidence-based practices [5]. The approach enables health service delivery points to work on cases with a chance of survival, allowing for open discussion and removing fear of blame among clients and healthcare providers [11]. Furthermore, it has proven to be a valuable metric for evaluating the quality of safe motherhood programs in populations [6].

The global estimated figure of near-miss in 2022 was 18.67/1000, with continental variations; 3.10/1000 in Europe to 31.88/1000 LB in Africa [12]. Socioeconomic factors (age, education level, wealth status), obstetric (parity, gravidity, history of CS delivery), medical conditions (having chronic hypertension), and health system-related characteristics were associated with MNM [13–17].

Although small-scale studies regarding MNM have been conducted within African countries, they were limited to subnational levels [13, 16–19] and with a relatively small sample size (e.g. *n* = 183 [20]). Therefore, large-scale studies are scarce to estimate MMN prevalence and risk factors across the continent. Furthermore, a recently conducted systematic review and meta-analysis on the global prevalence of MNM have not identified its risk factors did not estimate the pooled primary (direct and indirect) causes of MNM and have limited detailed evidence to understand the unique intervention options relevant to Africa [12]. This evidence gap could be partly addressed by synthesizing and pooling estimates from existing country-level evidence via systematic review methods and meta-analysis.

Hence, the current study aimed to assess the magnitude of MNM, its primary causes, and its potential determinants in Africa. This study's findings could aid in identifying factors that contribute to maternal morbidity and death, which is necessary for designing targeted measures aimed at improving maternal health outcomes, aligned with SDG target 3.1: reducing maternal mortality below 70 per 100,000 live births [21]. Policymakers, healthcare providers, and other stakeholders working in maternal health can use these findings to inform evidence-based decision-making and implement interventions, ultimately improving maternal health outcomes through strengthening targeted service quality measures.

## Methods and materials

### Study design and reporting system

A systematic review and meta-analysis were performed by synthesizing peer-reviewed articles. Preferred Reporting Items for Systematic Reviews and Meta-Analysis (PRISMA) was used to report the findings [22] (Table S1).

### Search strategies

This study considered studies published before October 31, 2023. Searches were performed from October 1–31, 2023 using electronic databases, namely PubMed/Medline, Scopus, Web of Science, Directory of Open Access Journals, and Google Scholar. Medical subject heading (MeSH) with Boolean operators (AND and OR) and truncation were employed to connect the keywords: maternal near miss, maternal morbidity, risk factors and Africa. A search strategy used for PubMed was: ((((((((epidemiology [All Fields]) OR (prevalence[All Fields])) OR (level[All Fields])) OR (magnitude[All Fields])) OR (proportion[All Fields])) OR (incidence[All Fields])) AND (((((((((maternal near miss[All Fields]) OR (maternal near-miss[All Fields])) OR (severe maternal outcome*[All Fields])) OR (pregnancy complication*[All Fields])) OR (life-threatening condition*[All Fields])) OR (maternal morbidit*[All Fields])) OR (Severe maternal complication*[All Fields])) OR (maternal mortality[All Fields])) OR (maternal death[All Fields]))) AND ((((determinant*[All Fields]) OR (factor*[All Fields])) OR (predictor*[All Fields])) OR (Associated factor*[All Fields]))) AND ((Africa*[All Fields]) OR (Sub-Saharan Africa*[All Fields])) Search strategies used across the database with their example are presented in the supplementary material (Table S2).

### Eligibility criteria and study selection

The systematic review and meta-analysis used the mnemonic Condition, Context, and Population (CoCoPop) for question formulation method [23].

Articles were included if they met the following inclusion criteria.

1. Condition (Co): Assessed the magnitude and/or determinants of MNM
2. Context(Co): Conducted in Africa
3. Population: All women who were pregnant, gave birth, or were within postpartum periods (42 days).
4. Study type: Observational (cross-sectional, case–control, and cohort) studies that reported the prevalence of MNM, its causes or determinants.

The scope of the review was limited to quantitative peer-reviewed published studies in the English language. The most complete and up-to-date study was included in case of duplicate studies sourced from the same data. Case reports, case series, commentaries, conference abstracts, letters to editors, technical reports, qualitative studies, and other opinion publications were excluded.

### Study selection, and data extraction

All retrieved studies were imported into the EndNote X7 library and checked for duplication. After removing duplicate studies, two independent reviewers (AH and YS) screened all articles for eligibility by looking at the title, abstract, and full text. A third reviewer (LL) independently assessed 20% of the excluded papers and collected the screened articles; any disagreements were resolved through discussion. Two authors (AH and YS) extracted the data by using Microsoft excel 2013 spreadsheet, which includes the author’s name, publication year, study year, study design, country, region, data collection technique, sample size, response rate, prevalence of MNM, each cause of MNM, and determinants.

### Quality assessment

The quality of the articles was assessed using the Joanna Briggs Institute (JBI) Critical Appraisal Checklist [24]. Two reviewers (AH and YS) independently rated the quality of the studies. The tool considers eight parameters, each with equal weight: (1) well-stated inclusion and exclusion criteria (2) a detailed description of study subjects and setting (3) measurement of exposures validly and reliably, (4) has well-stated objective with standard criteria used for measurement of the condition, (5) proper identification of confounders, (6) strategies to deal with confounders were well-stated (7), measurement of outcome validly and reliably and (8) use of appropriate statistical analysis. The evaluators rated the study a '1' if it met each specific parameter and a '0' if it did not (no or unclear). A composite index was computed and those studies with a score of ≥ 6 were included in the final analysis (SRMA) [25] (Table S3).

### Outcome measurement

MNM was assessed using the WHO MNM criteria and computed as the total number of MNM cases per total number of live births. MNM is defined as a woman admitted to health facilities with at least one of the following severe maternal complications: hypertensive disorders of pregnancy (severe preeclampsia or eclampsia), severe postpartum hemorrhage, uterine rupture, sepsis or severe systemic infection, or severe complications of abortion, but she survived [6]. Determinants of MNM were estimated using a pooled AOR with corresponding 95% CIs.

### Statistical analyses

Higgins I-square (I^2^) statistics and Cochran’s test were used to examine the presence of statistical heterogeneity across the included studies. Accordingly, considerable heterogeneity [I^2^ = 99.5%, *p* < 0.001] was detected, and the pooled prevalence of MNM and each severe maternal complication was estimated using a random-effects model with the DerSimonian-Laird method [26]. Furthermore, the adjusted odds ratio (AOR) and 95% CIs were extracted, and the pooled estimates were computed using a random- or fixed-effect model based on their level of heterogeneity. Forest plots were used to present a visual summary of data. In addition, subgroup analyses were performed based on region and study year.

A univariate meta-regression analysis with sample size, publication years, and study years as factors was performed to identify probable sources of heterogeneity among the studies [27]. Visual and statistical methods were used to check for publication biases. A funnel plot was used during the visual inspection, with a symmetrical and large inverted funnel used as a proxy for low publishing bias. In addition, statistical methods such as Egger's and Begg's tests were used to support visual assessment, *p*-value of < 0.05 suggests the possibility of publication bias. A random-effects model was used for the sensitivity analysis to examine the impact of a single study on the overall pooled prevalence of MNM.

## Results

### Study selection

**Fig. 1:**
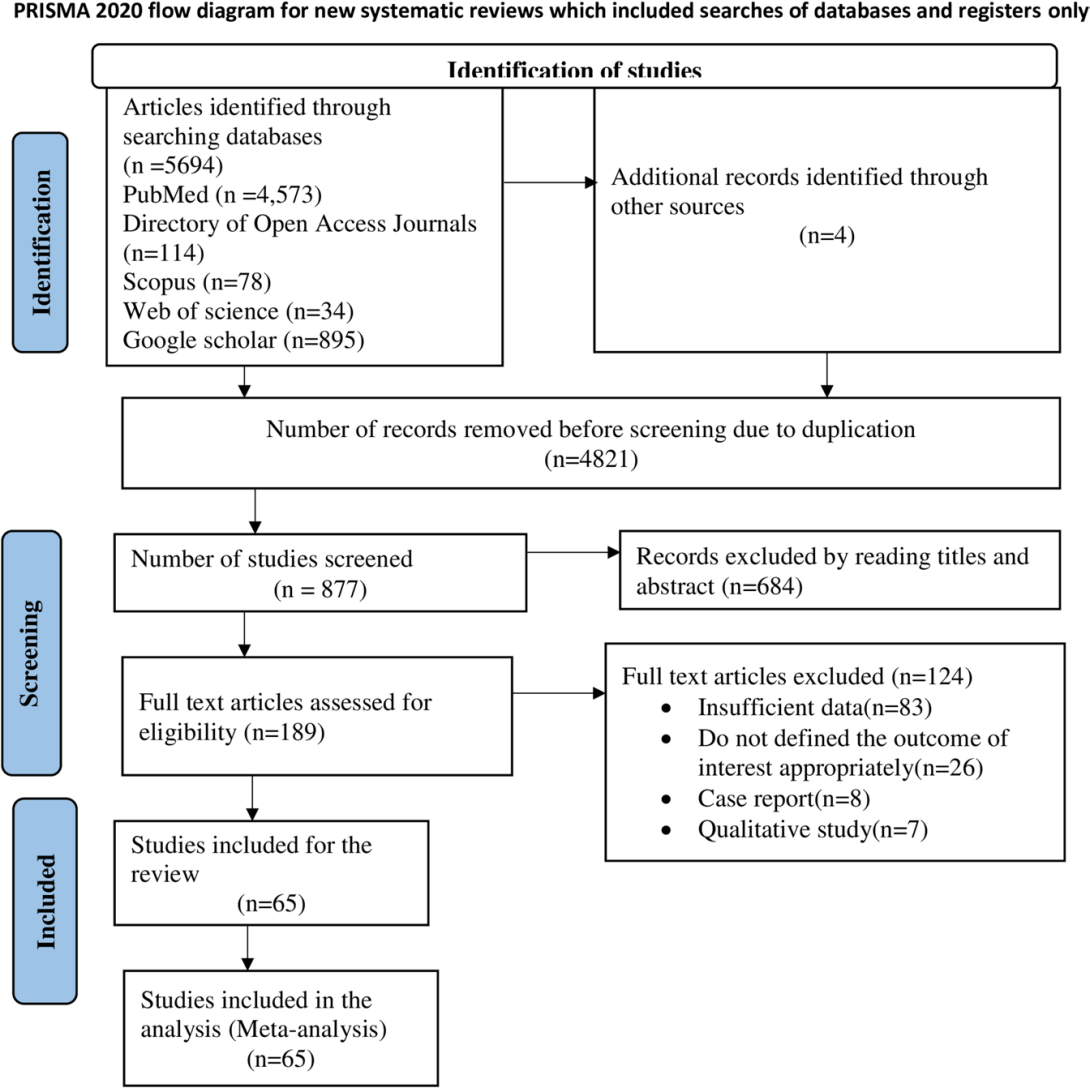
PRISMA flow diagram describing the selection of studies for systematic review and meta-analysis

Of 5698 retrieved studies, 4821 were duplicates (Fig. 1). Subsequently, 877 studies were reviewed by their titles and abstracts, with 189 articles meeting the full-text eligibility criteria. Sixty-five studies were included in this systematic review and meta-analysis. Most of the full-text reviewed articles were excluded (*n* = 124) due to not having insufficient data (*n* = 83), followed by failing to clearly state the outcome of interest (*n* = 26) (Fig. 1).

### Characteristics of included studies

In 65 studies, nearly one million (*N* = 968,555) participants were included, with the sample size in individual studies ranged from 183 [20] to 323,824 [28] women (Table 1). Nearly three-fourths (*n* = 47) of the studies were cross-sectional, and the remainder were case–control (*n* = 10) or cohort (*n* = 8) studies. The studies’ publication period spans from 2011 to 2023. Half of the studies (*n* = 33) were conducted by record review only. The majority of the studies were carried out in the East Africa (*n* = 43) and West Africa (*n* = 11) regions (Table 1).

**Table 1:** Descriptive summary of studies included in systematic review and meta-analysis of the prevalence of MNM and its determinants in Africa, 2008–2021

Study ID	Country	Region	Study year	Data collection methods	Sampling techniques	Sample size	Study participants	MNM-cases	MNMR/1000LB	Response rate	Quality
**Cross-sectional studies**											
Teka et al., 2022 [29]	Ethiopia	Eastern	2019	RR	C	5116	5116	146	28.0	100	7
Yemane and Tiruneh, 2020 [30]	Ethiopia	Eastern	2017	RR + I	SRS	845	845	210	248.5	100	7
Woldeyes et al.,2018 [31]	Ethiopia	Eastern	2015	I	C	2737	2737	138	59.0	100	8
Rysavy, 2023 [32]	Ethiopia	Eastern	2020	RR + I	C	658	658	70	11.0	100	7
Chikadaya et al., 2018 [14]	Zimbabwe	Eastern	2016	RR + I	C	11,871	11,871	110	92.6	100	8
Rulisa et al., 2015 [19]	Rwanda	Eastern	2012	RR + I	C	1739	1739	192	110.4	100	7
Gebremariam et al., 2022 [33]	Ethiopia	Eastern	2018	RR	SRS	941	905	129	143.0	96.2	8
Geleto et al., 2020 [28]	Ethiopia	Eastern	2015	RR	C	323,824	323,824	77,714	240.0	100	7
Ayele et al., 2014 [34]	Ethiopia	Eastern	2012	RR	C	8989	8989	206	23.0	100	8
Tenaw et al., 2021 [35]	Ethiopia	Eastern	2020	RR	C	1214	1214	108	88.9	100	8
Mekonnen et al., 2021 [36]	Ethiopia	Eastern	2019	RR + I	SRS	300	296	85	287.0	98.7	8
Worke et al., 2019 [37]	Ethiopia	Eastern	2018	I	SRS	572	572	152	266.0	100	7
Asaye, 2020 [38]	Ethiopia	Eastern	2019	RR + I	SRS	303	303	48	158.0	100	8
Dile and Seyum, 2015 [39]	Ethiopia	Eastern	2013	RR + I	SRS	806	806	188	233.0	100	8
Gedefaw et al., 2014 [40]	Ethiopia	Eastern	2013	RR	C	1355	1355	403	297.0	100	8
Wakgar et al., 2019) [41]	Ethiopia	Eastern	2016	RR	C	15,059	15,059	501	323.0	100	7
Alemu et al., 2019 [42]	South Sudan	Eastern	2016	RR	C	1041	1010	104	103.0	97.5	7
Ali et al., 2011 [43]	Sudan	Eastern	2008	RR	C	9578	9578	228	23.8	100	8
Nelissen et al., 2013 [44]	Tanzania	Eastern	2012	RR	C	9471	9136	216	23.6	96.5	7
Litorp et al., 2014 [45]	Tanzania	Eastern	2012	RR	C	13,121	13,584	467	35.6	96.6	8
Nansubuga et al., 2016 [46]	Uganda	Eastern	2013	I	SRS	1557	1557	434	278,7	100	8
Owolabi et al., 2020 [47]	Kenya	Eastern	2018	RR + I	srs	36,162	36,162	360	9.9	100	8
David et al., 2014 [48]	Mozambique	Eastern	2008	RR	C	27,916	27,916	564	20.2	100	7
Lilungulu et al., 2020 [49]	Tanzania	Eastern	2016	RR	C	3600	3480	124	35.6	96.67	7
Owolabi et al., 2017 [50]	Zambia	Eastern	2014	RR	C	2406	2406	392	162.9	100	7
Kachale et al., 2021 [16]	Malawi	Eastern	2017	RR	srs	458	458	161	352.0	100	8
Madouea et al., 2017 [51]	Chad	Middle	2016	RR	C	4857	4857	100	20.6	100	7
Foumsou et al., 2020 [52]	Chad	Middle	2019	RR	C	8124	8124	248	30.5	100	7
Chola et al., 2022 [53]	DRC	Middle	2019	RR	C	1390	1390	139	100.0	100	7
El-Agwany, 2019 [15]	Egypt	Northern	2015	RR	C	28,877	28,877	171	6.0	100	7
El Ghardallou et al., 2016 [54]	Tunisia	Northern	2010	RR	C	9957	9890	58	5.8	99.3	7
Abdel-Raheem et al.,2017 [55]	Egypt	Northern	2015	RR + I	SRS	17,503	17,503	342	19.5	100	7
Heitkamp et al.,, 2022 [56]	South Africa	Southern	2015	RR + I	C	32 161	32,161	379	11.7	100	7
Soma-Pillay et al., 2015 [57]	South Africa	Southern	2014	RR	C	26 614	26 614	117	4.4	100	7
Hlengani, 2019 [58]	South Africa	Southern	2015	RR	C	62,185	62,185	250	4.0	100	8
Heemelaar et al., 2020 [59]	Namibia	Southern	2019	RR	C	37 106	37 106	298	8.0	100	7
Heemelaar et al., 2019 [60]	Namibia	Southern	2018	RR	C	5772	5772	191	33.1	100	7
Drechsel et al., 2022 [61]	Ghana	Western	2020	RR + I	srs	447	447	148	331	100	7
Tunçalp et al., 2013 [62]	Ghana	Western	2011	RR	C	3438	3438	94	27.3	100	7
Sotunsa et al., 2019 [63]	Nigeria	Western	2013	RR	C	97 634	91,724	1451	15.3	93.94	7
Aduloju et al., 2018 [17]	Nigeria	Western	2016	RR	SRS	1897	1897	33	17.4	100	8
Adanikin et al., 2019 [64]	Nigeria	Western	2013	RR	C	5779	5779	366	63.0	100	7
Akpan et al., 2020 [65]	Nigeria	Western	2017	RR	C	10,111	10,111	691	68.3	100	7
Mbachu et al., 2017 [66]	Nigeria	Western	2015	RR + I	C	262	262	52	198.0	100	8
Adamu et al., 2019 [67]	Nigeria	Western	2013	RR	C	6753	6753	1451	215.0	100	7
Etuk et al., 2019 [68]	Nigeria	Western	2013	RR	C	100,107	91,724	2287	24.9	91.6	6
Oppong et al., 2019 [69]	Ghana	Western	2015	RR + I	C	8433	8433	288	34.1	100	7
Lori and Starke, 2012 [70]	Liberia	Western	2008	RR	C	750	750	120	160.0	100	7
**Cohort studies**											
Omona and Babirye, 2023 [71]	Uganda	Eastern	2019	RR	SRS	375	375	230	613.3	100	7
Nakimuli et al., 2016 [18]	Uganda	Eastern	2014	RR + I	C	3100	3100	695	224.0	100	8
Beyene et al., 2022 [72]	Ethiopia	Eastern	2018	RR + I	C	3010	2880	90	31.2	95.7	8
Tura et al., 2018 [73]	Ethiopia	Eastern	2017	RR + I	C	7404	7404	128	17.3	100	8
Kusheta et al., 2023 [74]	Ethiopia	Eastern	2019	RR + I	C	2724	2724	70	25.6	100	8
Kalisa et al., 2016 [75]	Rwanda	Eastern	2014	RR + I	C	3979	3979	86	21.6	100	8
Egal et al., 2022 [76]	Somalia	Eastern	2020	RR + I	C	6055	6055	342	51.3	100	8
Kebede et al., 2021 [77]	Ethiopia	Eastern	2014	RR + I	srs	1440	1440	96	67.0	100	8
**Case–control studies**											
Kasahun and Wako, 2018 [78]	Ethiopia	Eastern	2017	RR + I	SRS	229	229	77	_	100	7
Teshome et al., 2022 [79]	Ethiopia	Eastern	2020	RR + I	C	264	264	88	_	100	8
Danusa et al., 2022 [80]	Ethiopia	Eastern	2019	RR + I	srs	664	664	166	_	100	7
Dessalegn et al., 2020 [81]	Ethiopia	Eastern	2019	RR + I	C	321	321	80	_	100	7
Mekango et al., 2017 [82]	Ethiopia	Eastern	2016	RR + I	SRS	308	308	103	_	100	7
Habte and Wondimu,2021 [13]	Ethiopia	Eastern	2020	RR + I	SRS	322	322	81	_	100	7
Kumela et al., 2020 [20]	Ethiopia	Eastern	2018	I	srs	183	183	61	_	100	8
Dahie, 2022 [83]	Somalia	Eastern	2021	RR + I	C	533	533	178	_	100	7
Liyew et al., 2018 [84]	Ethiopia	Eastern	2016	RR + I	C	864	864	216	_	100	7
Total			2008–2021			984,034	968,555	95,511	9.86	98.4	

*MNMR/1000LB* Maternal Near-miss ratio per 1000 Live Births

Sampling techniques: *C* Consecutive sampling technique, *SRS* Systematic Random sampling, *srs* simple random sampling

Data collection methods: *RR* Record review, *RR* + *I* Record review and interview, *I* Interview

### The pooled estimate of MNM in Africa

**Fig. 2:**
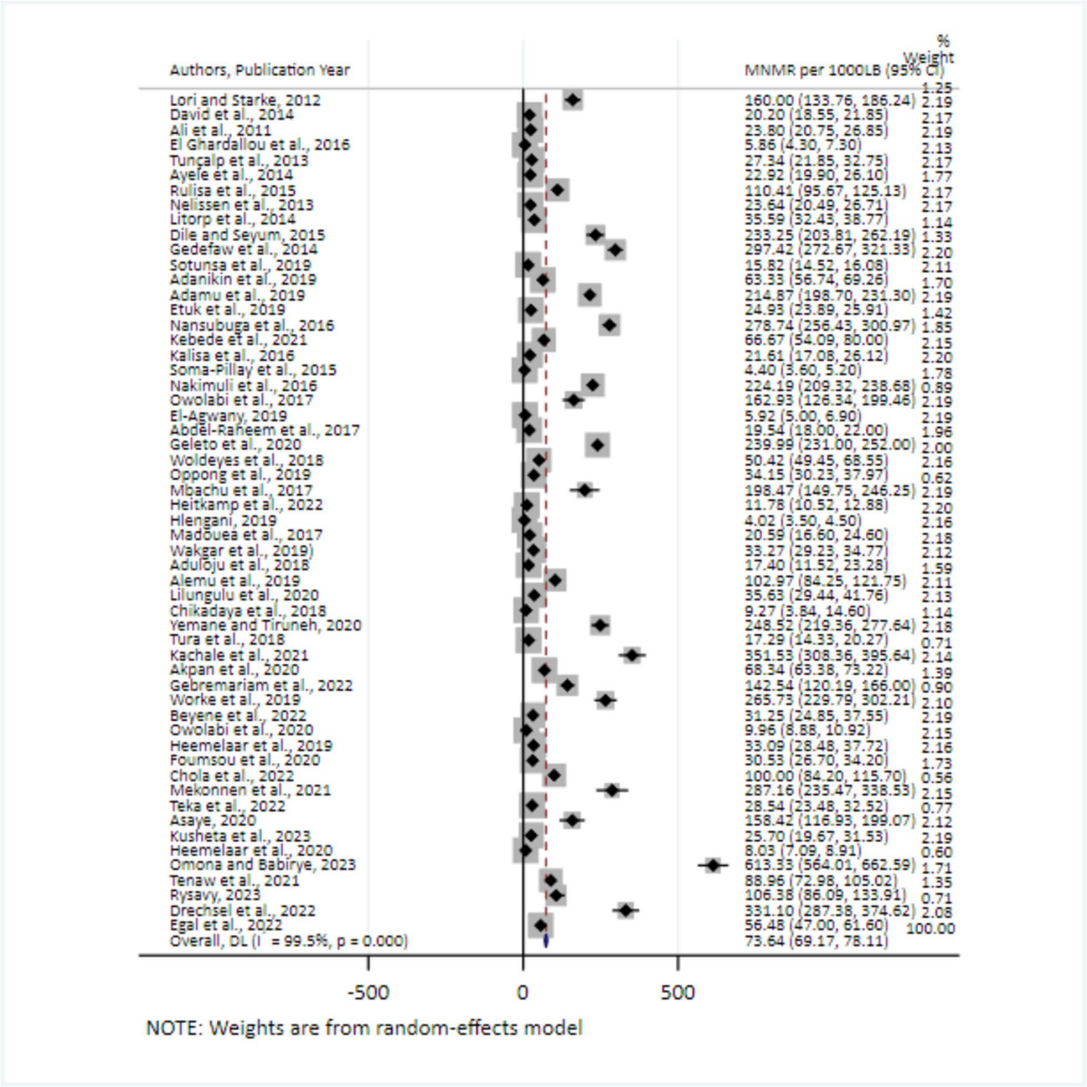
Forest plot showing the pooled estimates of MNMR in Africa, 2008–2021. The pooled prevalence of severe maternal complications among near-miss cases

The pooled estimate of MNM in Africa was 73.64/1000 Live births (95% CI: 69.17, 78.11) The I^2^ test statistic (I^2^ = 99.50%; *p* < 0.001) revealed that there was significant variation between the included studies (Fig. 2).

### Subgroup analyses

**Fig. 3:**
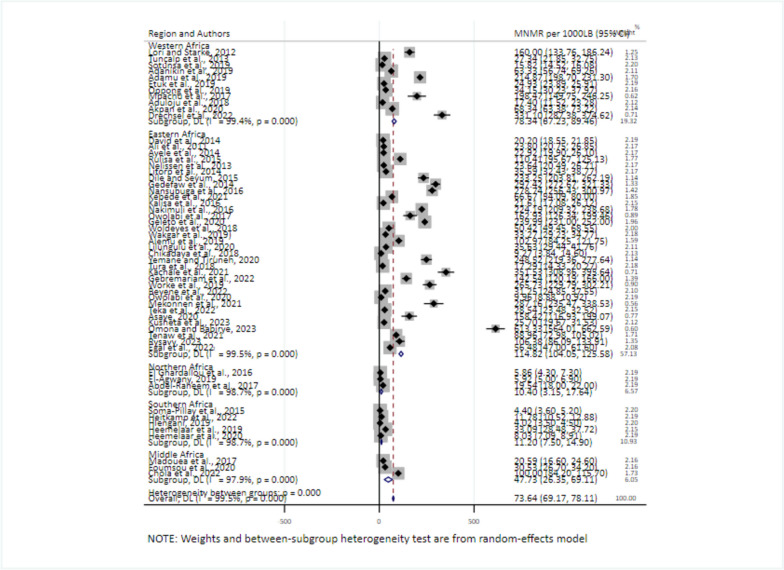
Sub-group analysis for the pooled prevalence of MNMR by regions of Africa, 2008–2021

Subgroup analyses by region, country, and study year were performed to examine the sources of variation in the pooled prevalence of MNM. East and West African regions have a higher pooled prevalence of MNM (114.82/1000LB (95% CI: 104.94, 123.59) and 78.34/1000LB (95% CI: 67.23, 89.46) respectively. In contrast, the Northern (10.40, 95% CI: 3.15, 17.64) and Southern (11.20, 95% CI: 7.5, 14.9) African regions had the lowest prevalence (Fig. 3).

**Fig. 4:**
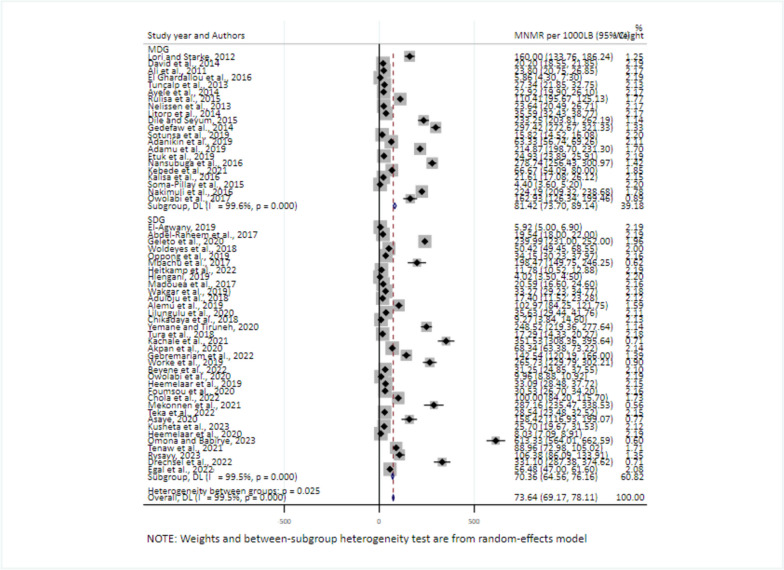
Sub-group analysis for the pooled prevalence of MNMR by study year in Africa, 2008–2021

For studies conducted before or during the Millennium Development Goals and during the SDG, the pooled prevalence was 81.42/1000 (95% CI: 73.70–89.14) and 70.36/1000 (95% CI: 64.56–76.16), respectively (Fig. 4).

### The pooled prevalence of severe maternal complications among near-miss cases

**Fig. 5:**
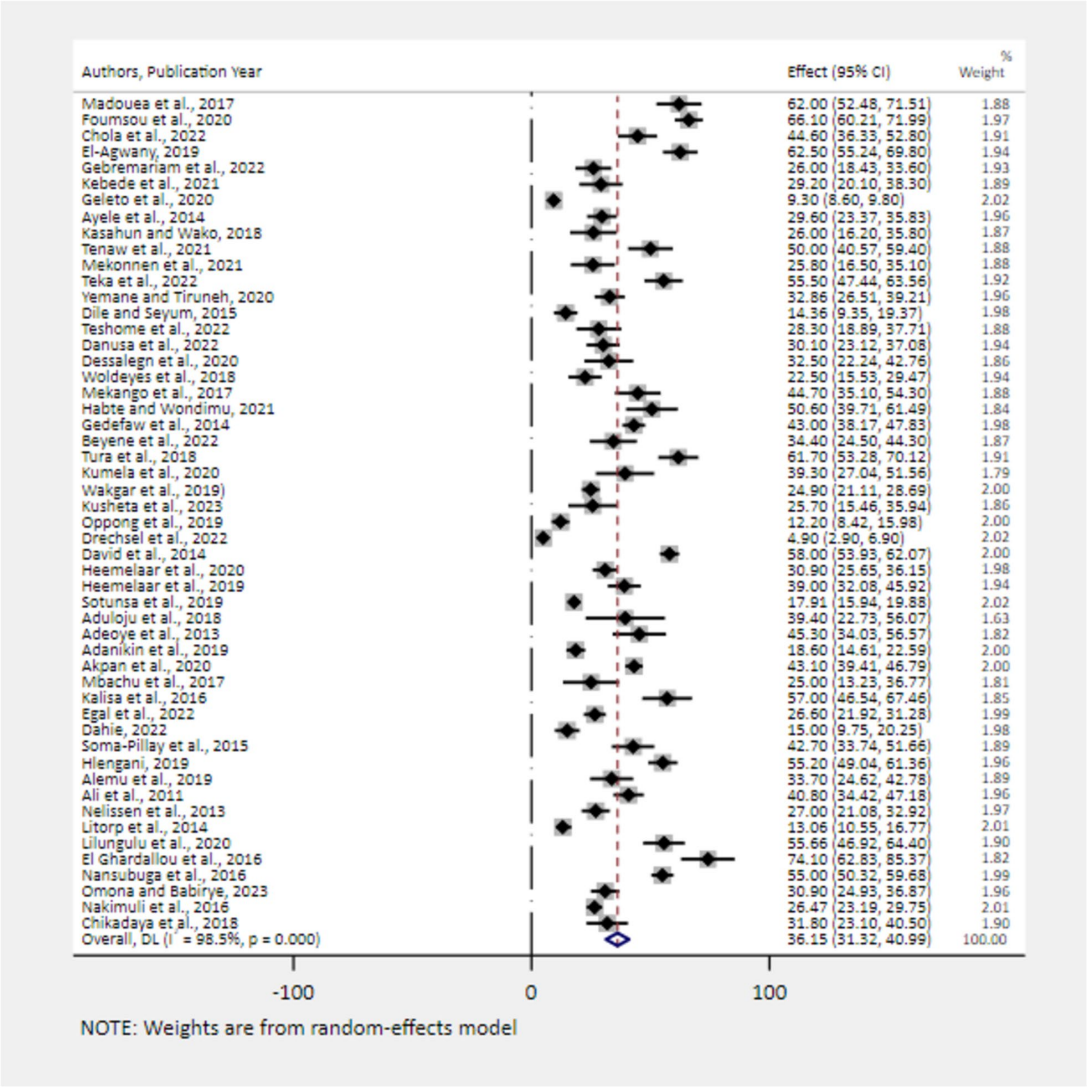
Forest plot showing the pooled prevalence of severe postpartum hemorrhage among near-miss cases in Africa, 2008–2021

**Fig. 6:**
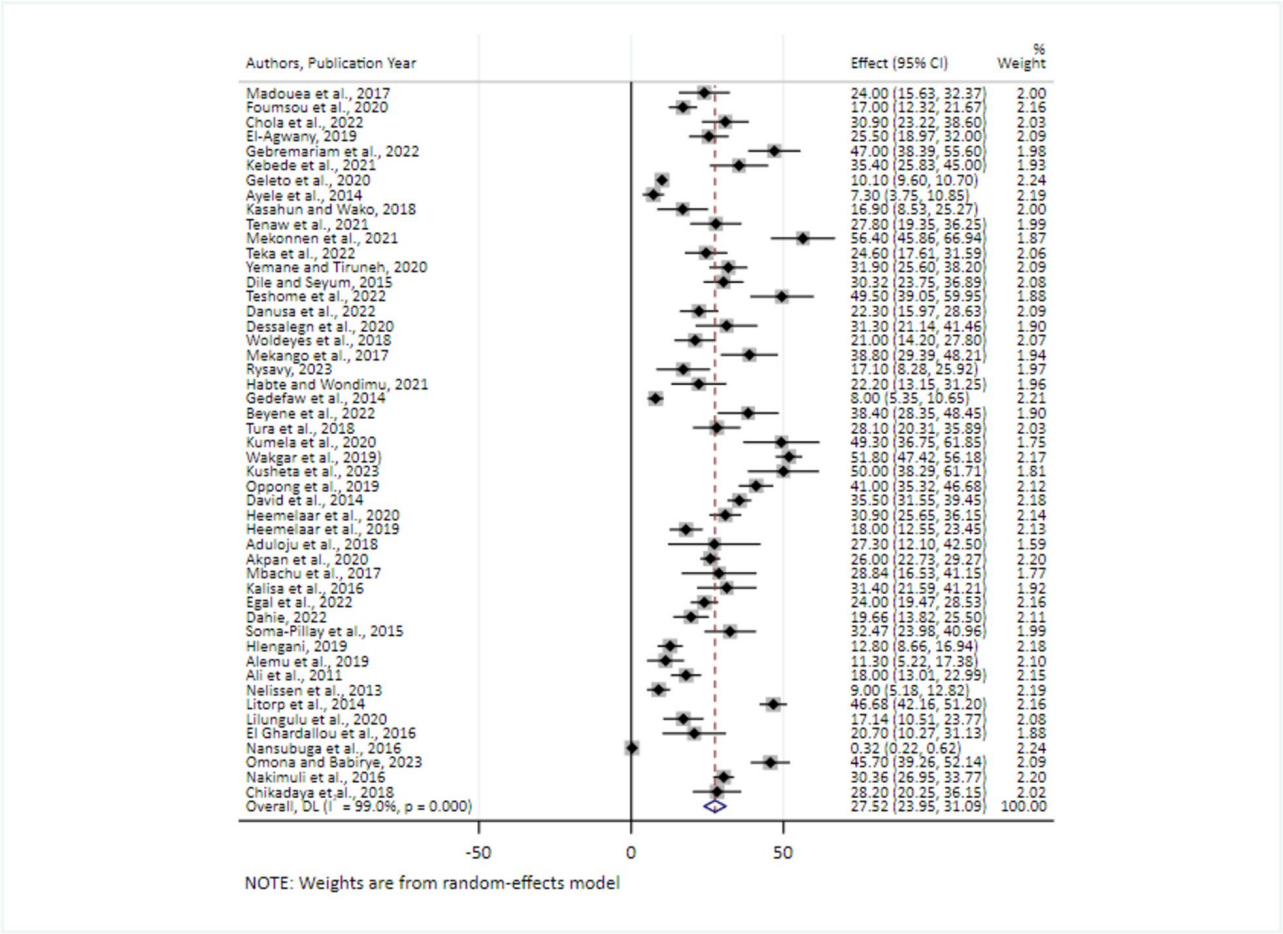
Forest plot showing the pooled estimates of severe forms of hypertension among near-miss cases in Africa, 2008–2021

**Fig. 7:**
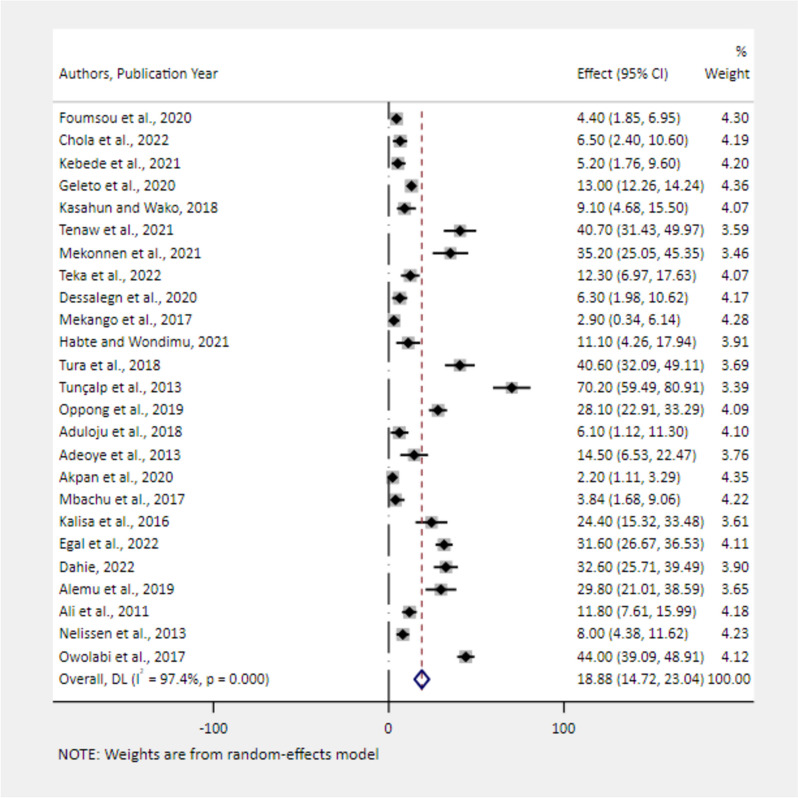
Forest plot showing the pooled estimates of severe anemia among near-miss cases in Africa, 2008–2021

**Fig. 8:**
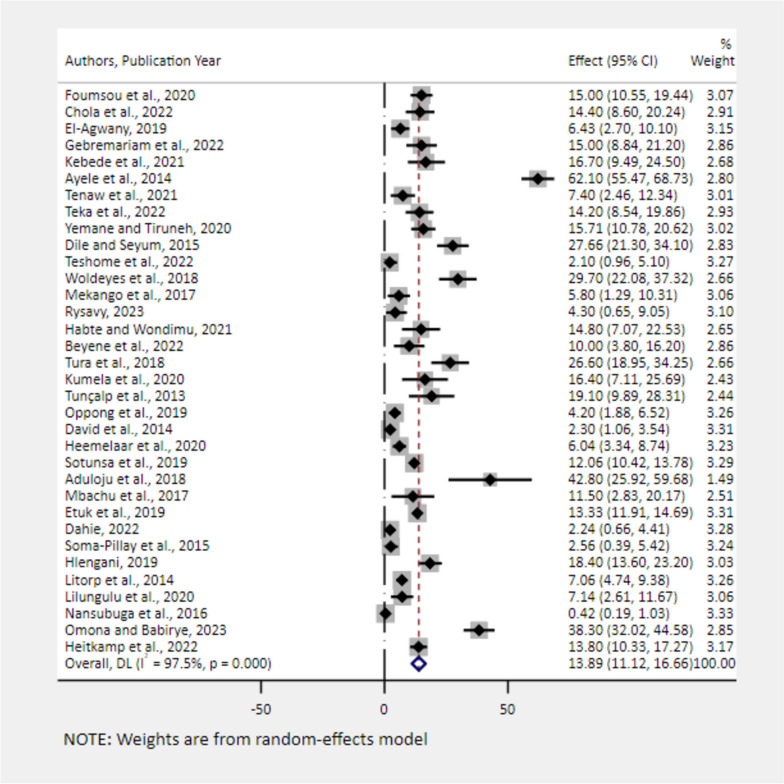
Forest plot showing the pooled estimates of uterine rupture among near-miss cases in Africa, 2008–2021

**Fig. 9:**
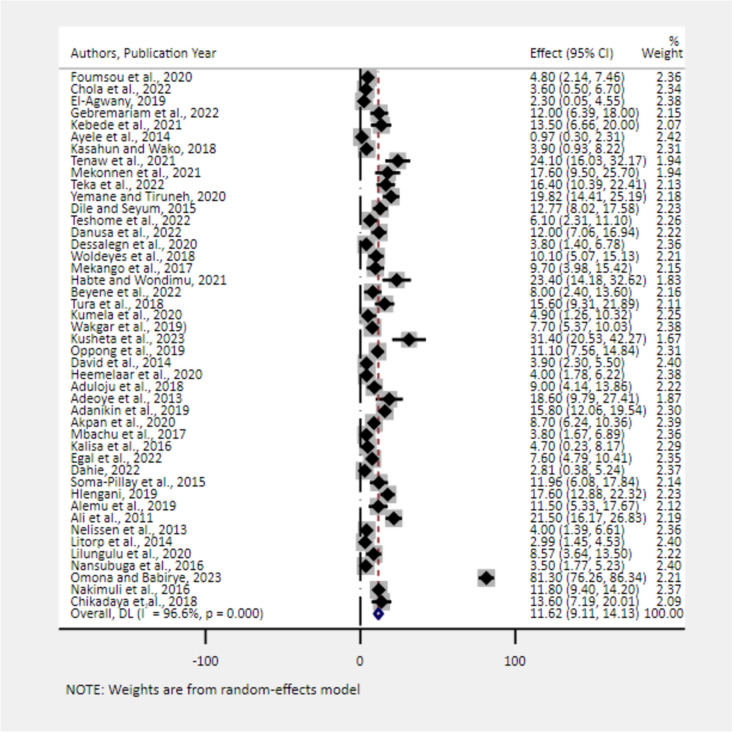
Forest plot showing the pooled estimates of sepsis among near-miss cases in Africa, 2008–2021

**Fig. 10:**
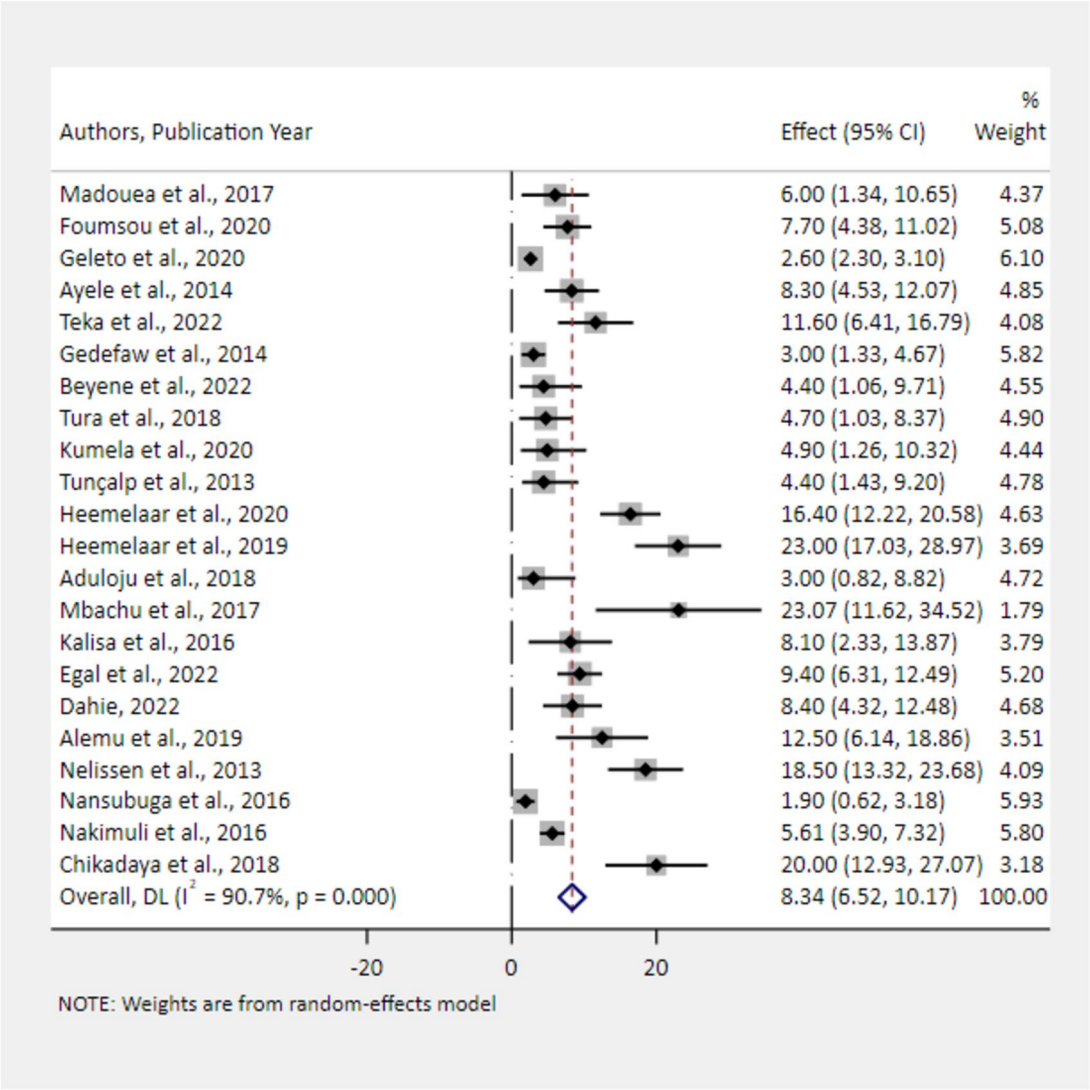
Forest plot showing the pooled estimates of abortion among near-miss cases in Africa, 2008–2021

The primary causes for being a near-miss case were severe postpartum haemorrhage (36.15%) (Fig. 5) and severe hypertension (27.52%) (Fig. 6). Severe anemia (18.88%) (Fig. 7), uterine rupture (13.89%) (Fig. 8), sepsis (11.62%) (Fig. 9), and septic abortion (8.34%) (Fig. 10) were also common severe maternal complications among the near-miss cases in Africa.

### Heterogeneity and publication bias

To determine the likely cause of heterogeneity, a univariate meta-regression analysis was performed using publication year, study year, and sample size. The sample size (*p* = 0.0074) substantially explained the heterogeneity, but significant heterogeneity was not observed by the study year (*p* = 0.421) or the publication year (*p* = 0.321) (Table 2).

**Table 2:** A univariate meta-regression analysis of factors affecting between-study heterogeneity, 2023

Heterogeneity source	Coefficients	SE	*p*-value	95% CI
Sample size	4.50E-07	0.036104	0.0074	3.54E-06, 2.3E-05
Publication year	.0036741	.0008883	0.321	-0.0019332, 0.0054151
Study year	.2845758	.0757447	0.421	0.1361189, 0.4330326

**Fig. 11:**
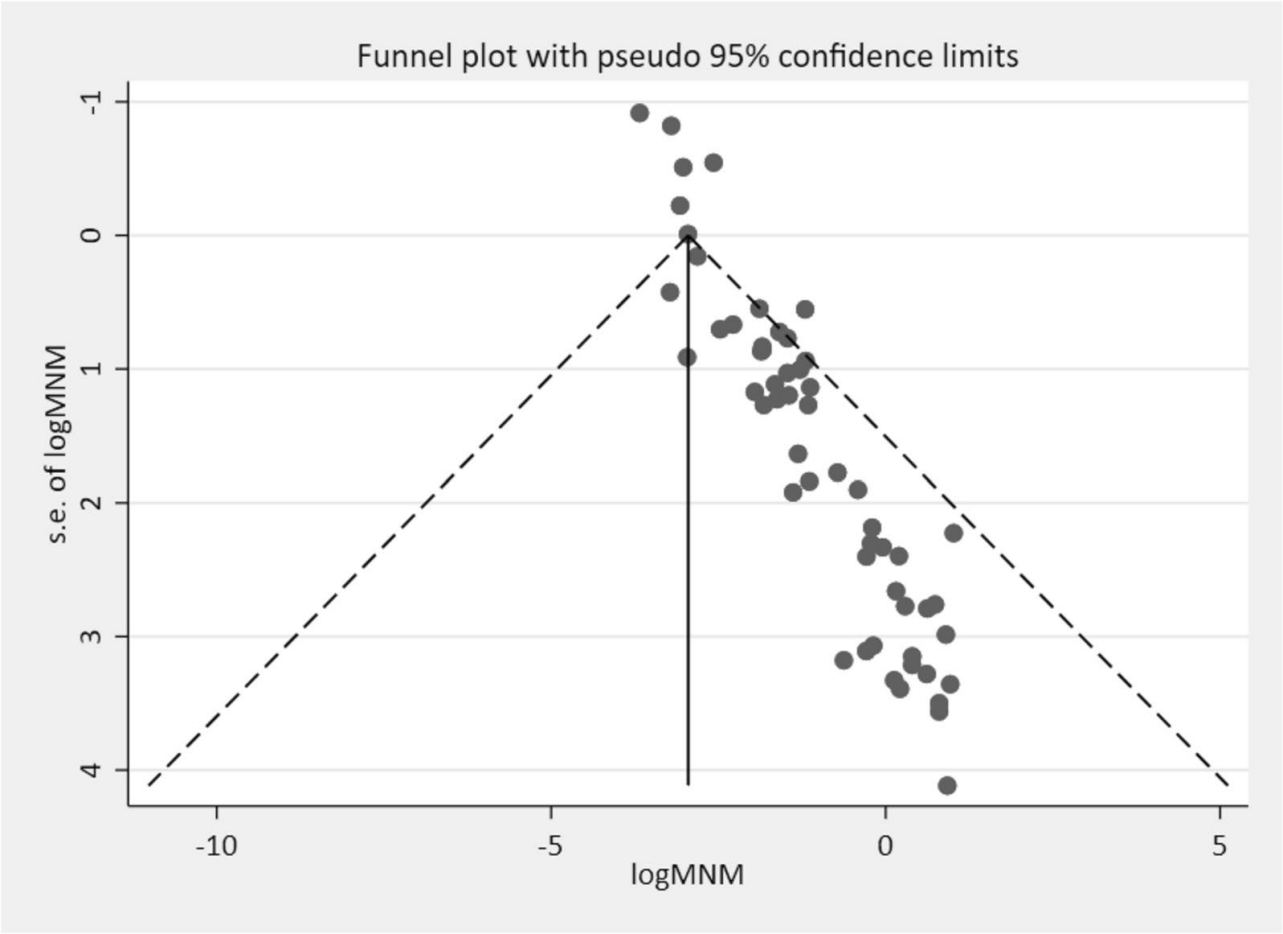
Funnel plot displaying publication bias of studies reporting the MNM in Africa, 2022

A funnel plot was used to examine publication bias visually, and the vast majority of studies were under an inverted funnel, indicating that publication bias was unlikely (Fig. 11). Furthermore, Egger's regression (*p* = 0.11) and adjusted Beggs rank correlation test (*p* = 0.11) did not show significant publication bias.

### Sensitivity analysis

**Fig. 12:**
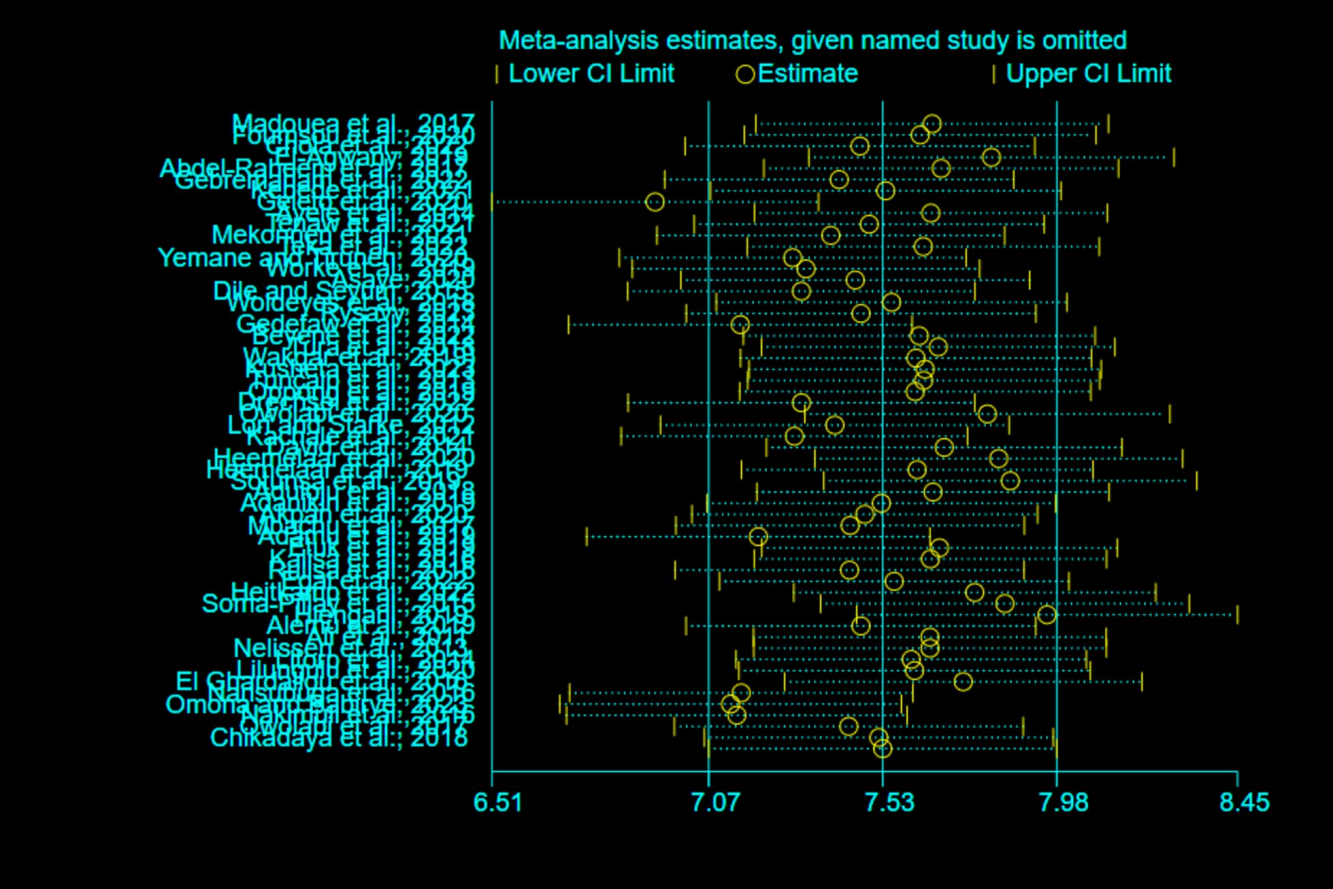
Sensitivity analysis for the pooled prevalence of MNM in Africa, 2008–2021

A sensitivity analysis using a random-effects model was carried out to detect the impact of a single study on the total meta-analysis estimate. There was no evidence that a single study had an effect on the overall prevalence of MNM (Fig. 12).

### Determinants of MNM in Africa

Nineteen variables were extracted from the included studies to identify determinants of MNM (S4 Excel). The risk of MNM was higher among women with advanced age, living in rural areas, low educational achievement, reported low ANC uptake, living far from a health facility, reported delay to access health service, and have previous history of CS or pre-existing medical condition (Table 3).

**Table 3:** Results of meta-analysis for significant determinants of MNM in Africa, 2008–2021

Variables	Authors	AOR(95% CI)	Weight	Heterogeneity (I^2^)
Age > 30 years	Tenaw et al., 2021 [35]	2.29(1.22, 4.29)	5.90	17.13%
	Kachale et al., 2021 [16]	3.14(1.09, 9.020)	0.88	
	Aduloju et al., 2018 [17]	1.95(1.50, 2.30)	86.87	
	Dahie, 2022 [83]	2.72(1.60, 4.56)	6.35	
	Overall	**2.03(1.65, 2.40)**	**100**	
Residence (rural)	Kebede et al.,2021 [77]	1.68(1.01, 2.78)	18.43	47.3%
	Yemane and Tiruneh, 2020 [30]	2.16(1.34, 3.50)	14.96	
	Liyew et al.,2018 [84]	10.60(4.59, 24.46)	0.31	
	Rysavy, 2023 [32]	3.710(2.23, 6.17)	6.40	
	Gedefaw et al.,2014 [40]	2.10(1.40, 3.10)	19.14	
	Aduloju et al., 2018 [17]	1.48(1.08, 1.98)	28.46	
	Dahie, 2022 [83]	2.68(1.70, 4.23)	12.31	
	Overall	**2.06(1.50, 2.61)**	100	
Low monthly income	Worke et al., 2019 [37]	2.85(1.43, 5.55)	54.38	13.12%
	Asaye, 2020 [38]	3.99(1.65, 9.65)	14.42	
	Dahie, 2022 [83]	3.33(1.15, 10.53)	10.50	
	Alemu et al., 2019 [42]	3.01(1.16, 7.84)	20.69	
	Overall	**3.09(1.58, 4.62)**	100.00	
Long distance	Yemane and Tiruneh, 2020 [30]	2.27(1.33, 3.86)	27.12	55.2%
	Danusa et al.,2022 [80]	4.02(1.82, 8.89)	3.44	
	Mekango et al., 2017 [82]	2.80(1.19, 6.35)	6.48	
	Rysavy, 2023 [32]	11.93(5.20, 27.39)	0.35	
	Habte and Wondimu, 2021 [13]	3.21(1.61, 6.39)	7.55	
	Gedefaw et al., 2014 [40]	1.90(1.17, 2.94)	55.06	
	**Overall**	**2.26(1.61, 2.92)**	**100**	
No formal education	Dile and Seyum, 2015 [39]	2.00(1.09, 3.69)	12.61	37.03%
	Teshome et al., 2022 [79]	4.80(1.78, 12.90)	0.69	
	Danusa et al., 2022 [80]	3.06(1.31, 7.13)	2.52	
	Dessalegn et al., 2020 [81]	2.24(1.17, 4.31)	2.52	
	Mekango et al., 2017 [82]	3.20(1.24, 8.12)	1.80	
	Aduloju et al., 2018 [17]	1.58(1.20, 2.30)	70.44	
	Dahie, 2022 [83]	2.83(1.26, 6.34)	3.30	
	Overall	**1.82(1.36, 2.28)**	100.00	
No ANC	Tenaw et al., 2021 [35]	3.04(1.58, 5.83)	0.58	16.9
	Yemane and Tiruneh, 2020 [30]	1.65(1.13, 2.55)	5.20	
	Worke et al., 2019 [37]	3.16(1.96, 5.10)	1.07	
	Dile and Seyum, 2015 [39]	2.51(1.50, 4.20)	1.44	
	Teshome et al., 2022 [79]	2.75(1.13, 6.72)	0.34	
	Danusa et al.,2022 [80]	2.25(1.10, 4.61)	0.85	
	Liyew et al., 2018 [84]	5.58(1.94, 16.07)	0.05	
	Dessalegn et al.,2020 [81]	3.71(1.10, 12.76)	0.08	
	Woldeyes et al.,2018 [31]	1.92(1.09, 3.45)	1.90	
	Habte and Wondimu, 2021 [13]	3.25(2.21, 7.69)	0.35	
	Aduloju et al., 2018 [17]	1.73(1.53, 1.88)	85.79	
	Adeoye et al., 2013 [4]	5.26(2.70, 11.11)	0.15	
	Dahie, 2022 [83]	2.68(1.82, 4.00)	2.20	
	**Overall**	**1.80(1.64, 1.97)**	**100.0**	
1st Delay	Abdel-Raheem et al., 2017 [55]	3.43(1.54, 7.52)	5.80	25.03%
	Worke et al., 2019 [37]	1.99(1.10, 3.61)	32.92	
	Dile and Seyum, 2015 [39]	4.02(2.34, 6.90)	9.98	
	Dessalegn et al., 2020 [81]	5.74(2.93, 11.20)	3.03	
	Woldeyes et al.,2018 [31]	2.37(1.36, 4.12)	27.23	
	Adeoye et al., 2013 [4]	2.07(1.03, 4.17)	21.04	
	**Overall**	**2.51(1.79, 3.23)**	**100**	
2nd delay	Abdel-Raheem et al., 2017 [55]	2.51(1.11, 5.68)	9.02	7.03%
	Dile and Seyum, 2015 [39]	3.85(2.11, 7.03)	7.83	
	Woldeyes et al., 2018 [31]	2.66(1.39, 5.070)	13.61	
	Dahie, 2022 [83]	1.77(1.21, 2.59)	69.54	
	**Overall**	**2.12(1.42, 2.82)**	**100.0**	
3rd delay	Abdel-Raheem et al., 2017 [55]	3.12(1.28, 7.69)	21.12	69.9%
	Yemane and Tiruneh, 2020 [30]	1.56(1.03, 2.34)	37.75	
	Dile and Seyum, 2015 [39]	7.02(3.89, 12.65)	15.10	
	Woldeyes et al., 2018 [31]	4.12(2.34, 7.26)	26.04	
	**Overall**	**3.38(1.21, 5.55)**	**100.0**	
Previous history of CS	Kasahun and Wako, 2018 [78]	7.68(3.11, 18.96)	1.31	12.92%
	Tenaw et al., 2021 [35]	4.48(2.67, 7.53)	13.93	
	Teshome et al., 2022 [79]	3.70(1.42, 9.60)	4.92	
	Dessalegn et al., 2020 [81]	3.53(1.49, 8.36)	6.97	
	Mekango et al.2017 [82]	4.60(1.98, 7.61)	10.38	
	Habte and Wondimu, 2021 [13]	3.53(1.79, 6.98)	12.21	
	Kachale et al., 2021 [16]	4.08(2.34, 7.09)	14.58	
	Adeoye et al., 2013 [4]	3.72(1.93, 14.90)	1.96	
	Omona and Babirye, 2023 [71]	3.74(2.35, 5.91)	25.96	
	Heitkamp et al., 2022 [56]	8.40(5.80, 12.30)	7.79	
	**Overall**	**4.35(3.44, 5.26)**	**100**	
Presence of any medical condition	Tenaw et al., 2021 [35]	3.13(1.57, 6.26)	11.47	25.78%
	Asaye, 2020 [38]	5.13(2.08, 12.60)	2.28	
	Liyew et al., 2018 [84]	10.80(5.16, 22.60)	0.83	
	Dessalegn et al., 2020 [81]	2.04(1.11, 3.78)	35.38	
	Mekango et al., 2017 [82]	3.05(1.78, 6.93)	9.51	
	Habte and Wondimu, 2021 [13]	2.79(1.45, 5.37)	16.41	
	Adeoye et al., 2013 [4]	6.85(1.96, 23.93)	0.52	
	Heitkamp et al., 2022 [56]	2.40(1.10, 5.10)	15.76	
	Oppong et al., 2019 [69]	5.95(3.75, 9.42)	7.84	
	**Overall**	**2.91(2.12, 3.71)**	100	

The effect of age on being a near-miss case was identified in four studies [16, 17, 35, 83], with the pooled risk of being a near-miss case was 2.03 times higher among women aged 30 years and above than women aged < 30 years [AOR = 2.03; 95%CI: 1.65, 2.40)]. From pooled estimates of seven studies, being a rural resident was associated with MNM [17, 30, 32, 40, 77, 83, 84]; women from rural areas were 2.06 times more likely to be near-miss cases than urban counterparts [AOR = 2.06; 95%CI: 1.50, 2.61)]. Using the data of seven studies [17, 39, 79–83], the overall likelihood of MNM was 1.82 times higher among women with no formal education [AOR = 1.82; 95%CI: 1.36, 2.28]. Thirteen studies [4, 13, 17, 30, 31, 35, 37, 39, 79–81, 83, 84] were selected to assess the pooled association between not receiving ANC and MNM, and women who did not receive ANC were 1.80 times more likely to become near miss cases than women who did receive ANC [AOR = 1.80; 95%CI: 1.64, 1.97]. A pooled estimate from ten studies [4, 13, 16, 35, 56, 71, 78, 79, 81, 82] revealed that women with a previous history of CS were 4.35 times more likely to have MNM than their counterparts[AOR = 4.35; 95%CI: 3.44, 5.26]. All three (1st, 2nd, and 3rd) delays were significantly associated with MNM. The odds of MNM were 2.51 [AOR = 2.51; 95% CI: 1.79, 3.23], 2.12[AOR = 2.12; 95% CI: 1.42, 2.82], and [AOR = 3.38; 95% CI: 1.21, 5.55] times higher among women who experienced 1st, 2nd and 3rd delays respectively. Long distance to health facilities and low monthly income were also identified as significant predictors of MNM in Africa (Table 3).

## Discussion

The pooled prevalence of MNM was 73.77/1000 live births, which varied significantly across the regions and study periods. The risk of MNM was higher among women with advanced age, living in rural areas, low educational achievement, reported low ANC uptake, living far from a health facility, reported delay to access health service, and have previous history of CS or pre-existing medical condition.

The current finding of MNM in Africa (73.77/1000 live births) was considerably higher than the global estimate (18.67/1000LB) [12]. This could be attributed to a lack of access to adequate healthcare services, road infrastructure and transportation access limitations, ill-equipped health facilities, socioeconomic inequities, low educational achievement and high fertility rate, all of which are prevalent across the continent [85–87]. The pooled prevalence of MNM was higher in the East and West African regions. Compared to the northern and southern sub-regions of Africa, these two regions are known for poor healthcare infrastructure [88, 89], low skilled birth attendance rates [90], poverty and lack of education, a high rate of harmful traditional practices such as female genital mutilation [91], and political and social instability, all of which contribute to poor maternal health outcomes.

Furthermore, there has been a decrease in prevalence of MNM since 2015 (during the SDG era) compared to that before 2015 (during the MDG era). This, might be attributed to the implementation of SDG goal 3: ensuring healthy lives and promoting well-being for all. In particular, Goal 3.1 focuses on the global reduction of the maternal mortality ratio through great investment and effort to address complications that contribute to MNM [92]. In addition, governments emphasize the significance of establishing robust and resilient health systems during the SDG by providing skilled maternal health services such as prenatal, skilled delivery and postnatal services, which are vital for preventing and managing problems that can lead to MNM [93, 94]. Moreover, it could be attributed to technological breakthroughs and enhanced healthcare interventions, increasing global awareness and advocacy for maternal health, and a focus on women's empowerment.

Women who did not receive adequate ANC had a higher likelihood of being near-miss cases, which is consistent with the previous studies [95–97]. Timely and adequate ANC entails regular check-ups and monitoring of maternal and fetal health, along with counselling about danger signs and the need to obtain healthcare when needed [98]. In addition, ANC provides preventive services (vaccination, iron and folic acid supplementation, and mother-to-child HIV transmission prevention) as well as screening for risk factors such as hypertension and diabetes [98, 99]. If these check-ups, counselling, preventive services, and screening are not provided as part of regular ANC follow-ups, these problems may go unnoticed and untreated, increasing the likelihood of a near miss. Moreover, ANC is often linked to planning for skilled birth attendance, as part of the birth preparedness and complication readiness (BPCR) plan [100]. Thus, a lack of ANC could lower the likelihood of accessing skilled delivery services, increase the risk of complications during childbirth, and limit access to emergency obstetric care, all of which increase the risk of severe maternal outcomes. Thus, efforts should be made to ensure universal access to ANC for a positive pregnancy experience by addressing barriers to accessing healthcare services for pregnant women, improving the healthcare system, and promoting educational campaigns to improve maternal and neonatal outcomes.

The current findings regarding the higher risk of MNM among women with three delays of service use were supported by previous studies [101–103]. These three delays refer to a framework used in maternal health to identify and address factors contributing to MNM [104]. An expectant mother who experiences the first delay (delay at home), the second delay (delay on the road to the health facility), and the third delay (delay at the health facility) could experience greater difficulties by delaying timely care during pregnancy and childbirth [103–106]. The possible reasons behind those delays are being unaware of danger signs, delayed decision-making, lack of transportation, and ill-equipped health system. Thus, African governments need to work together to address all three delays through community education, better infrastructure construction, and improved care quality.

Women with a history of Caesarean section were at a higher risk of experiencing MNM, which is in line with previous studies conducted in Brazil [96], India [107], and Thailand [108]. Caesarean section (CS) is a life-saving intervention for the fetus, mother, or both at the time of life-threatening conditions such as obstructed labor, fetal distress, and obstetric hemorrhage [108]. However, deliveries after previous CS have been reported to have a higher risk of adverse pregnancy outcomes. This could be because scar tissue from previous CS can complicate subsequent deliveries by causing uterine rupture and antepartum hemorrhage (due to placenta previa and placenta accreta) [109–111]. This study implies that when evaluating the clinical grounds for CS, healthcare providers ought to weigh its potential risk over its benefits (especially in the case of elective CS) and may consider alternative birthing options when appropriate. On the other hand, healthcare personnel should pay special attention to women with a history of CS during prenatal and intrapartum care.

Similarly, women with pre-existing medical conditions had a higher risk of developing MNM, in line with similar studies [4, 101, 112, 113]. This might be due to chronic medical conditions, such as hypertension or diabetes, which can lead to life-threatening complications during pregnancy, such as preeclampsia, gestational diabetes, or worsening of an existing medical condition [112, 113]. In addition, these medical disorders might impair the immune system [114], leaving pregnant women more susceptible to infections, which, if not treated effectively and promptly, can lead to severe maternal outcomes.

Background characteristics, such as lack of formal education, rural residence, low monthly income, and distance from health facilities, were also identified as significant predictors of MNM. Previous studies have supported these findings [115, 116]. A possible explanation could be that those women have limited access to healthcare services and may need to travel far to reach health facilities, which might result in delays in receiving essential maternity care [13]. Furthermore, they may have limited access to maternal healthcare, which might result in delayed detection and management of complications that lead to MNM. Thus, a comprehensive approach is needed to ensure universal access to maternal healthcare for women in hard-to-reach areas by improving healthcare infrastructure and promoting community awareness.

This study has both strengths and limitations. This is the first systematic review and meta-analysis in Africa to examine the pooled prevalence of MNM and its contributing factors. In addition, the number and the quality of articles that have been meta-analysis are high, reflecting a comprehensive view of MNM. Furthermore, this study revealed primary severe maternal problems that resulted in MNM. Thus, the findings could be used as input for stakeholders in Africa who work on reducing maternal mortality and morbidities. However, the findings should be interpreted in light of the following limitations. First, since the vast majority of the included studies were hospital-based and the data collection techniques relied on record review, the findings may not be generalizable to near-misses that were not present at service delivery points. Furthermore, as the majority of the articles were from Eastern, Western, and Southern African regions, this may raise the issue of generalizability.

## Conclusion

The prevalence of MNM was 73.77/1000 live births, with higher rates reported in eastern, western, and middle African countries. The risk of MNM increased among women living in rural areas, possessing low income, not attended formal education, not received ANC, living far from health facilities, reported three delays in seeking health service, have a previous history of CS, and had pre-existing medical conditions. A comprehensive approach is needed to strengthen and ensure universal access to education and maternal health services, especially ANC, to women in hard-to-reach areas by improving healthcare infrastructure and promoting community awareness. Stakeholders should work together to tackle all three delays through community education and awareness campaigns, improve access to road infrastructure and transportation, and improve the quality of care provided at service delivery points.

### Supplementary Information

**Additional file 1: Table S1. **PRISMA 2020 Checklist for the systematic review and meta-analysis on the pooled estimate of maternal near-miss, its primary causes and determinants in Africa.

**Additional file 2: Table S2. **Examples of searching strategy for systematic review and meta-analysis on the pooled estimate of maternal near-miss, its primary causes, and determinants in Africa, 2023.

**Additional file 3: Table S3.** JBI Critical Appraisal Checklist for analytical cross-sectional studies used for assessing the individual quality of studies included in the systematic review and meta-analysis, 2023.

**Additional file 4. **

## Data Availability

No datasets were generated or analysed during the current study.

## Abbreviations

AOR: Adjusted odds ratio
ANC: Antenatal Care
CS: Caesarean Delivery
MNM: Maternal Near-Miss
MNMR: Maternal Near-Miss Ratio
PRISM: Preferred Reporting Items for Systematic Reviews and Meta-Analysis
SDG: Sustainable Development Goal
