# Supplementary material for: Road to maternal death: the pooled estimate of maternal near-miss, its primary causes and determinants in Africa: a systematic review and meta-analysis
Source: BMC Pregnancy Childbirth. 2024 Feb 17;24:144. doi: 10.1186/s12884-024-06325-1 (PMC10874058; doi:10.1186/s12884-024-06325-1)
Supplement: Supplementary file 2 — Additional file 2: Table S2. Examples of searching strategy for systematic review and meta-analysis on the pooled estimate of maternal near-miss, its primary causes, and determinants in Africa, 2023. [file 12884_2024_6325_MOESM2_ESM.docx]

**TableS2.** Examples of searching strategy for systematic review and meta-analysis on the pooled estimate of maternal near-miss, its primary causes, and determinants in Africa, 2023

| **Database** | **Example of searching strategy** | **Number of studies** |
| --- | --- | --- |
| PubMed | ((((((((epidemiology [All Fields]) OR (prevalence[All Fields])) OR (level[All Fields])) OR (magnitude[All Fields])) OR (proportion[All Fields])) OR (incidence[All Fields])) AND (((((((((maternal near miss[All Fields]) OR (maternal near-miss[All Fields])) OR (severe maternal outcome*[All Fields])) OR (pregnancy complication*[All Fields])) OR (life-threatening condition*[All Fields])) OR (maternal morbidit*[All Fields])) OR (Severe maternal complication*[All Fields])) OR (maternal mortality[All Fields])) OR (maternal death[All Fields]))) AND ((((determinant*[All Fields]) OR (factor*[All Fields])) OR (predictor*[All Fields])) OR (Associated factor*[All Fields]))) AND ((Africa*[All Fields]) OR (Sub-Saharan Africa*[All Fields])) | 4573 |
| Google Scholar | allintitle: Maternal near miss severe OR maternal OR outcome OR complications OR pregnancy OR childbirth OR postpartum OR Africa "Maternal near miss" -Asia, -europe -America | 791 |
| Directory of Open Access Journals | 'Maternal near miss ' OR 'sever maternal outcome' OR Morbidit* | 114 |
| Scopus | TITLE-ABS-KEY ( '' Maternal Near-miss” AND Africa | 78 |
| Web of science | TOPIC: (Maternal Near-miss and Africa) | 34 |
