# Supplementary material for: Road to maternal death: the pooled estimate of maternal near-miss, its primary causes and determinants in Africa: a systematic review and meta-analysis
Source: BMC Pregnancy Childbirth. 2024 Feb 17;24:144. doi: 10.1186/s12884-024-06325-1 (PMC10874058; doi:10.1186/s12884-024-06325-1)
Supplement: Supplementary file 3 — Additional file 3: Table S3. JBI Critical Appraisal Checklist for analytical cross-sectional studies used for assessing the individual quality of studies included in the systematic review and meta-analysis, 2023. [file 12884_2024_6325_MOESM3_ESM.docx]

**TableS3.** JBI Critical Appraisal Checklist for analytical cross-sectional studies used for assessing the individual quality of studies included in the systematic review and meta-analysis, 2023.

| Author name, Year of Publication | The sampling frame appropriate to address the target population | The study subjects and the setting described in detail | Study participants sampled in an appropriate way | Study subjects and the setting described in detail | Data analysis conducted with sufficient coverage of the identified sample | Valid methods used for the identification of the condition | Reliability of the instrument used to measure the condition | Appropriateness of Statistical analysis methods | Total | Risk of bias |
| --- | --- | --- | --- | --- | --- | --- | --- | --- | --- | --- |
| Omona and Babirye, 2023 | 1 | 1 | 1 | 1 | 1 | 0 | 1 | 1 | 7 | Low |
| Nakimuli et al., 2016 | 1 | 1 | 1 | 1 | 1 | 1 | 1 | 1 | 8 | Low |
| Beyene et al., 2022 | 1 | 1 | 1 | 1 | 1 | 1 | 1 | 1 | 8 | Low |
| Tura et al., 2018 | 1 | 1 | 1 | 1 | 1 | 1 | 1 | 1 | 8 | low |
| Kusheta et al., 2023 | 1 | 1 | 1 | 1 | 1 | 1 | 1 | 1 | 8 | Low |
| Kalisa et al., 2016 | 1 | 1 | 1 | 1 | 1 | 1 | 1 | 1 | 8 | Low |
| Egal et al., 2022 | 1 | 1 | 1 | 1 | 1 | 1 | 1 | 1 | 8 | Low |
| Kebede et al., 2021 | 1 | 1 | 1 | 1 | 1 | 1 | 1 | 1 | 8 | Low |
| Teka et al., 2022 | 1 | 1 | 1 | 1 | 1 | 0 | 1 | 1 | 7 | Low |
| Yemane and Tiruneh, 2020 | 1 | 1 | 0 | 1 | 1 | 1 | 1 | 1 | 7 | Low |
| Woldeyes et al., 2018 | 1 | 1 | 1 | 1 | 1 | 1 | 1 | 1 | 8 | low |
| Rysavy, 2023 | 1 | 1 | 1 | 1 | 1 | 0 | 1 | 1 | 7 | Low |
| Chikadaya et al., 2018 | 1 | 1 | 1 | 0 | 1 | 1 | 1 | 1 | 8 | Low |
| Rulisa et al., 2015 | 1 | 1 | 0 | 1 | 1 | 1 | 1 | 1 | 7 | Low |
| Gebremariam et al., 2022 | 1 | 1 | 1 | 1 | 1 | 1 | 1 | 1 | 8 | Low |
| Geleto et al., 2020 | 1 | 1 | 0 | 1 | 1 | 1 | 1 | 1 | 7 | Low |
| Ayele et al., 2014 | 1 | 1 | 1 | 1 | 1 | 1 | 1 | 1 | 8 | Low |
| Tenaw et al., 2021 | 1 | 1 | 1 | 1 | 1 | 1 | 1 | 1 | 8 | Low |
| Mekonnen et al., 2021 | 1 | 1 | 1 | 1 | 1 | 1 | 1 | 1 | 8 | Low |
| Worke et al., 2019 | 1 | 1 | 1 | 1 | 1 | 0 | 1 | 1 | 7 | low |
| Asaye, 2020 | 1 | 1 | 1 | 1 | 1 | 1 | 1 | 1 | 8 | Low |
| Dile and Seyum, 2015 | 1 | 1 | 1 | 1 | 1 | 1 | 1 | 1 | 8 | Low |
| Gedefaw et al., 2014 | 1 | 1 | 1 | 1 | 1 | 1 | 1 | 1 | 8 | Low |
| Wakgar et al., 2019) | 1 | 1 | 1 | 1 | 1 | 0 | 1 | 1 | 7 | Low |
| Alemu et al., 2019 | 1 | 1 | 1 | 1 | 1 | 0 | 1 | 1 | 7 | Low |
| Ali et al., 2011 | 1 | 1 | 1 | 1 | 1 | 1 | 1 | 1 | 8 | Low |
| Nelissen et al., 2013 | 1 | 1 | 1 | 1 | 1 | 0 | 1 | 1 | 7 | low |
| Litorp et al., 2014 | 1 | 1 | 1 | 1 | 1 | 1 | 1 | 1 | 8 | Low |
| Nansubuga et al., 2016 | 1 | 1 | 1 | 1 | 1 | 1 | 1 | 1 | 8 | Low |
| Owolabi et al., 2020 | 1 | 1 | 1 | 1 | 1 | 1 | 1 | 1 | 8 | Low |
| David et al., 2014 | 1 | 1 | 0 | 1 | 1 | 1 | 1 | 1 | 7 | Low |
| Lilungulu et al., 2020 | 1 | 1 | 0 | 1 | 1 | 1 | 1 | 1 | 7 | Low |
| Owolabi et al., 2017 | 1 | 1 | 0 | 1 | 1 | 1 | 1 | 1 | 7 | Low |
| Kachale et al., 2021 | 1 | 1 | 1 | 1 | 1 | 1 | 1 | 1 | 8 | low |
| Madouea et al., 2017 | 1 | 1 | 0 | 1 | 1 | 1 | 1 | 1 | 7 | Low |
| Foumsou et al., 2020 | 1 | 1 | 0 | 1 | 1 | 1 | 1 | 1 | 7 | Low |
| Chola et al., 2022 | 1 | 1 | 0 | 1 | 1 | 1 | 1 | 1 | 7 | Low |
| El-Agwany, 2019 | 1 | 1 | 1 | 1 | 1 | 0 | 1 | 1 | 7 | Low |
| El Ghardallou et al., 2016 | 1 | 1 | 0 | 1 | 1 | 1 | 1 | 1 | 7 | Low |
| Abdel-Raheem et al., 2017 | 1 | 1 | 1 | 1 | 1 | 0 | 1 | 1 | 7 | Low |
| Heitkamp et al., 2022 | 1 | 1 | 0 | 1 | 1 | 1 | 1 | 1 | 7 | low |
| Soma-Pillay et al., 2015 | 1 | 1 | 0 | 1 | 1 | 1 | 1 | 1 | 7 | Low |
| Hlengani, 2019 | 1 | 1 | 1 | 1 | 1 | 0 | 1 | 1 | 8 | Low |
| Heemelaar et al., 2020 | 1 | 1 | 1 | 1 | 1 | 0 | 1 | 1 | 7 | Low |
| Heemelaar et al., 2019 | 1 | 1 | 0 | 1 | 1 | 1 | 1 | 1 | 7 | Low |
| Drechsel et al., 2022 | 1 | 1 | 0 | 1 | 1 | 1 | 1 | 1 | 7 | Low |
| Tunçalp et al., 2013 | 1 | 1 | 1 | 1 | 1 | 0 | 1 | 1 | 7 | Low |
| Sotunsa et al., 2019 | 1 | 1 | 0 | 1 | 1 | 1 | 1 | 1 | 7 | low |
| Aduloju et al., 2018 | 1 | 1 | 1 | 1 | 1 | 1 | 1 | 1 | 8 | Low |
| Adanikin et al., 2019 | 1 | 1 | 1 | 1 | 1 | 0 | 1 | 1 | 7 | Low |
| Akpan et al., 2020 | 1 | 1 | 0 | 1 | 1 | 1 | 1 | 1 | 7 | Low |
| Mbachu et al., 2017 | 1 | 1 | 1 | 1 | 1 | 1 | 1 | 1 | 8 | Low |
| Adamu et al., 2019 | 1 | 1 | 1 | 1 | 1 | 0 | 1 | 1 | 7 | Low |
| Etuk et al., 2019 | 1 | 1 | 0 | 1 | 1 | 0 | 1 | 1 | 6 | Low |
| Oppong et al., 2019 | 1 | 1 | 1 | 1 | 1 | 0 | 1 | 1 | 7 | low |
| Lori and Starke, 2012 | 1 | 1 | 1 | 1 | 1 | 0 | 1 | 1 | 7 | Low |
| Kasahun and Wako, 2018 | 1 | 1 | 1 | 1 | 1 | 0 | 1 | 1 | 7 | Low |
| Teshome et al., 2022 | 1 | 1 | 1 | 1 | 1 | 1 | 1 | 1 | 8 | Low |
| Danusa et al., 2022 | 1 | 1 | 1 | 1 | 1 | 0 | 1 | 1 | 7 | Low |
| Dessalegn et al., 2020 | 1 | 1 | 1 | 0 | 1 | 1 | 1 | 1 | 7 | Low |
| Mekango et al., 2017 | 1 | 1 | 1 | 1 | 1 | 0 | 1 | 1 | 7 | low |
| Habte and Wondimu, 2021 | 1 | 1 | 1 | 1 | 1 | 0 | 1 | 1 | 7 | Low |
| Kumela et al., 2020 | 1 | 1 | 1 | 1 | 1 | 1 | 1 | 1 | 8 | Low |
| Dahie, 2022 | 1 | 1 | 1 | 0 | 1 | 1 | 1 | 1 | 7 | Low |
| Liyew et al., 2018 | 1 | 1 | 1 | 0 | 1 | 1 | 1 | 1 | 7 | Low |
